# On the Thermal Performance of a Fractal Microchannel Subjected to Water and Kerosene Carbon Nanotube Nanofluid

**DOI:** 10.1038/s41598-020-64142-w

**Published:** 2020-04-29

**Authors:** Zongjie Lyu, Farzad Pourfattah, Ali Akbar Abbasian Arani, Amin Asadi, Loke Kok Foong

**Affiliations:** 1 Institute of Research and Development, Duy Tan University, Da Nang, 550000 Vietnam; 2 Faculty of Civil Engineering, Duy Tan University, Da Nang, 550000 Vietnam; 3 Department of Mechanical Engineering, University of Kashan, Kashan, Iran; 4 Department for Management of Science and Technology Development, Ton Duc Thang University, Ho Chi Minh City, Vietnam; 5 Faculty of Civil Engineering, Ton Duc Thang University, Ho Chi Minh City, Vietnam

**Keywords:** Mechanical engineering, Computational nanotechnology

## Abstract

Using single layer microchannels accompanied by nanofluids is one of the most practical solutions in thermal management of high power density devices. The main challenge in cooling systems of electronic devices is to provide a uniform temperature distribution. In the present study, fluid flow and heat transfer in a fractal microchannel heatsink have been simulated employing the computational fluid dynamics (CFD) method. The fractal microchannel is used to achieve uniform temperature distribution. Thermal performance of single-walled carbon nanotubes (SWCNT) and multi-walled carbon nanotubes (MWCNT) dispersed in the two base fluids of water and kerosene in a fractal microchannel at Reynolds (Re) numbers of 1500 to 3000 are investigated. It should be noted that the nanofluids have been simulated by the two-phase mixture model. The results indicated that the use of fractals silicon microchannel leads to having a uniform temperature distribution. Based on the results, at maximum Re number when the working fluid is water, Nu number and pumping power are 20.9 and 0.033 W whereas, in kerosene flow at the same condition, Nu number and pumping power are 6 and 0.054 W, respectively. According to the obtained results, using the SWCNT nanoparticle compared with the MWCNT nanoparticle leads to a significant enhancement in the Nusselt (Nu) number. This difference is more pronounced by increasing the Re number and nanoparticle volume fraction. In addition, the results indicated that at the same Re number and nanoparticle volume fraction, the performance evaluation criterion of the water-based nanofluid is 4 times higher than that of the kerosene-based nanofluid. So the use of the water as the working fluid with the SWCNT nanoparticle for cooling in the fractal silicon microchannel is recommended.

## Introduction

Nowadays, the increase in heat transfer rate in various applications and devices, especially high-power density electronic devices, has become an important issue^1–4^. With the introduction of nanofluid by Choi and Eastman^5^ in 1995, its applications in various industries have been widely recognized. Moreover, many researchers have studied the behavior of these new heat transfer materials and fluids^6–12^. Due to the importance of the topic, many researchers have reviewed the published literature on different aspects of nanofluids, such as preparation methods^13,14^, thermophysical properties^15,16^, heat transfer applications^17,18^, and different simulation techniques^19,20^. Moreover, with the advent of microchannels, new doors in thermal management in various industries, especially in the electronics industry, has been opened^21–25^. Many researchers combine new methods of enhancing heat transfer, especially the use of nanofluids, to increase the heat transfer rate^26,27^. Changes in geometric conditions, including the channels arrangement, cross-sectional changes, and composition of microchannels, resulted in a significant improvement in heat transfer performance. Among the new microchannel structures, one can refer to fractal microchannels^23,28^. In this type of microchannel, the fractal pattern of the circulatory and respiratory system of mammals has been adapted. Due to the practical applications of this structure, many researchers have studied the properties of flow and heat transfer in this structure. Many researchers employed computational fluid dynamics (CFD) as a powerful tool to simulate the fluid flow and heat transfer of different fluids^29–31^, including nanofluids. Ghodoossi^32^ investigated laminar and turbulent flow in the fractal microchannel network. Hydrodynamic and thermal analysis shows that a tree-like fractal network in microchannel heat sinks enhances the hydrodynamic performance. This investigation showed different results in laminar and turbulent flow regimes base on hydrodynamic performance relative to the classical parallel microchannel heat sinks. Liu *et al*.^33^ studied a microchannel heat sink T-Y type numerically and experimentally. They found that the heat transfer coefficient and the pressure drop in the numerical investigation is consistent with experimental data. Their result has shown that the optimal bifurcation angle is 60°, and as a cooling fluid has a more favorable thermal performance than water. Xu *et al*.^34^ studied the effect of pulsation on the flow and heat transfer in the fractal silicon microchannel network using numerical simulation and experimental data. They presented local Nusselt Number (Nu) distribution, temperature distribution, and pressure loss in different Reynolds numbers (Re). Their result indicated that the heat transfer in the pulsation frequency range of 0–40 Hz and Re number from 1,800 to 2,800 is enhanced compared with the steady flow where the enhancement factors diminish with increasing the Re number. Zhang *et al*.^35^ numerically studied the fluid flow and heat transfer in the fractal-like microchannel networks. They also investigated the effects of the secondary flow and the flow circulation at the bifurcations of the microchannel. Their results revealed that the flow rate and aspect ratio of the channels significantly enhanced the vortices evolution in the fractal-like microchannel, which results in enhancing the fluid mixing and heat transfer efficiency. Zhang *et al*.^36^ numerically and experimentally investigated the effects of S-type and straight fractal-like microchannel on the fluid flow and heat transfer characteristic. They compared the results of experiments with those of simulations employing the conventional equivalent and subsectional integral methods and found that the developed subsectional integral method has better performance to predict the friction factor and Nusselt number compared to other methods. The effects of different flow field configuration in a mini-channel heat sink on the temperature uniformity have been numerically studied by Mu *et al*.^37^. They analyzed the effect of various parameters on temperature uniformity. They reported that the temperature non-uniformity decreased to less than 1 K under the heat flux of 2 MWm^−2^ on a 3 × 3 cm heating surface.

In another study, the fluid flow and heat transfer performance of steam in a fractal tree-like microchannel has been numerically and experimentally studied by Shui *et al*.^38^. Their results revealed that the studied microchannel showed lower friction factor under the turbulent flow regime. Moreover, better heat transfer efficiency has also been achieved by applying a high Re number and heat flux. Chen *et al*.^39^ performed an experimental and numerical study to investigate the flow field and heat transfer in the Y and T bifurcation microchannel. They indicate that the Y-bifurcation microchannel provided significantly better heat transfer performance than that of existing T-bifurcation microchannels.

Using nanoparticles in the base fluid in order to increase mixing in the flow path and the porous medium has attracted many researchers′ attention. Pourfattah *et al*.^40^ inspected inclined rectangular rib attack angle effect on the turbulent heat transfer of a water-Al_2_O_3_ nanofluid in a tube. They presented pressure loss and heat transfer in different inclined rectangular rib attack angle for three volume fraction of Al_2_O_3_ nanoparticle in the water. Their investigation showed that the fluid-thermal performance coefficient, heat transfer increment to the pressure drop ratio in the rib with the attack angle equal to 60° is maximum.

Carbon nanotubes (CNTs) have special thermophysical properties with very high thermal conductivity ^25,27,41–46^. As it is shown in previous investigations^47–53^, carbon nanotube in any type (SWCNT, MWCNT) has a great influence on the thermophysical properties of nanofluids, fluid flow, and, heat transfer. Combining the effect of using fractal and nanofluid containing carbon nanotube in microchannel results in an interesting finding in heat transfer performance and temperature distribution. Based on the referred idea, in the present numerical study, the fluid flow and heat transfer of a nanofluid in a 3D fractal microchannel were examined. The 3D fractal microchannel is investigated numerically and experimentally by Xu *et al*.^54^ for the pure water. In this research, temperature distribution and heat transfer efficiency in the fractal microchannel for Re numbers of 1,500-3,000 are studied. To improve heat transfer, nanofluid made from water and kerosene fluids with carbon nanotubes were investigated. Investigating the flow and temperature fields of the nanofluids (water and kerosene-based) in the fractal silicon microchannel and analysis of the temperature distribution, pressure drop, Nu number, PEC, temperature, and pressure distribution at each investigated Re numbers, distinguish the current study from other similar studies.

## Problem statement and numerical model

### Fractal microchannel model

**Figure 1:**
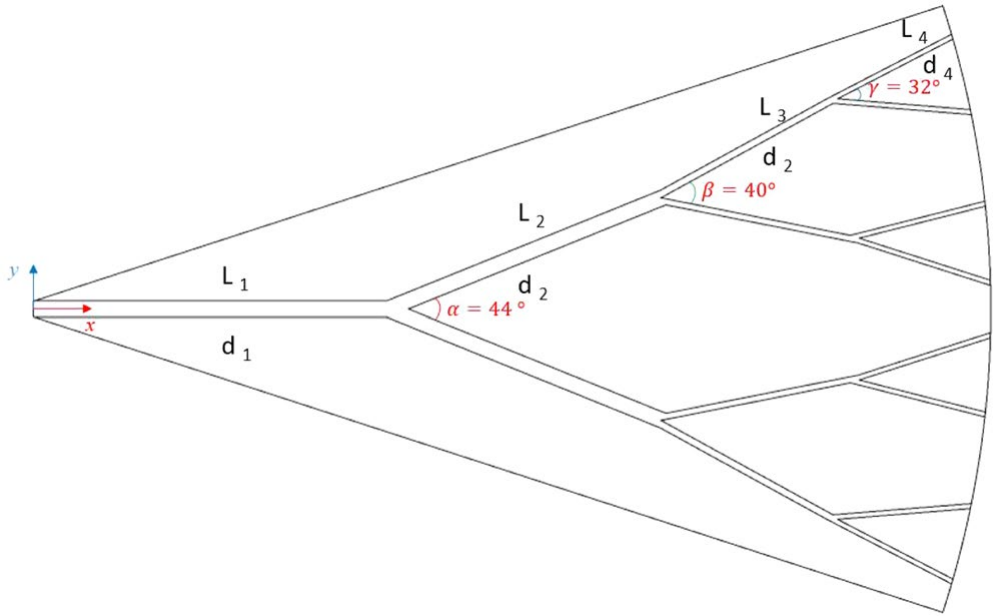
The schematic geometry of the studied fractal microchannel.

Using fractal microchannels, due to their large number of applications in many natural patterns such as the fractal system of the blood circulation and respiration of mammals, would be an operational structure for the cooling of electronic devices. In the present study, the water and kerosene were considered as the base fluid. Three-dimensional flow and heat transfer of water and kerosene with SWCNT and MWCNT nanofluid in different solid volume fractions ranging from 0-8 *vol*. % and different Re numbers ranging from 1500–3000 in a fractal silicon microchannel have been numerically simulated. The main objective of this research is to study the fluid flow and temperature distribution of nanofluids in the fractal microchannel and to analyze the parameters such as Nu number, performance efficiency coefficient (PEC), temperature distribution, and pressure drop. Figure 1 presents a schematic geometry of fractal microchannel. Each of the dimensions d_1_, d_2_, d_3_, d_4,_ and L_1_, L_2_, L_3_, L_4_ are the diameters and lengths of the different branches through the inlet to outlet sections of the fractal microchannel. The path of each microchannel branch is separated by different angles; α = 44°, β = 40°, and γ = 32°. All the dimensions are provided in Table 1.

**Table 1:** Geometrical dimensions of the studied fractal microchannel.

Length (mm)	Hydraulic diameter (mm)		
L_1_	8.01	d_1_	0.286
L_2_	5.66	d_2_	0.277
L_3_	4.00	d_3_	0.180
L_4_	2.83	d_4_	0.413

### Boundary Conditions

**Figure 2:**
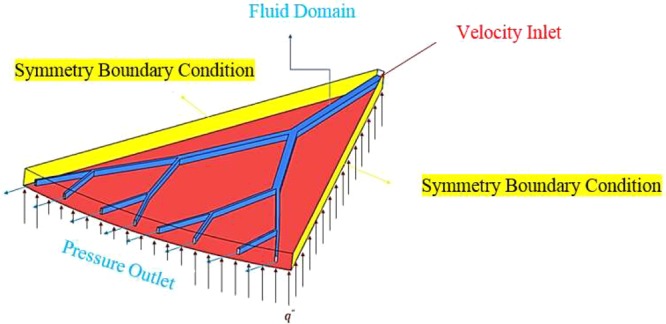
Boundary conditions.

In the present study, fluid flow and heat transfer in a fractal microchannel have been numerically simulated employing the finite volume method, which is a widely used method by researchers^55,56^. The applied boundary conditions in the computation domain have been presented in Fig. 2. Since it is a symmetrical structure, to reduce the time and cost of the computational field, the symmetry boundary condition is used in the external boundary regions (yellow areas in Fig. 2). Applying inlet velocity boundary condition, the fluid enters the microchannel at the temperature of 293 *K* and uniform velocity (red arrow in Fig. 2). The material of fractal microchannel is silicon, and it is under the constant heat flux boundary condition at q′′ = 40 *W/cm*^2^ (the lower red area in Fig. 2). The outlet of each branch of the fractal microchannel is influenced by the atmospheric pressure with the outlet pressure boundary condition. Moreover, the fluid domain in the silicon substrate is shown in blue. The silicon substrate thickness is 0.75 *mm*. Moreover, the embedded microchannels are located precisely in the middle of the thickness. The no-slip boundary condition is valid on the walls of the microchannel^57,58^.

### Thermophysical properties of nanofluids

In this research, water (Pr = 6.2) and Kerosene (Pr = 21) are considered as the base fluids, and SWCNT and MWCNT nanoparticles have been used as the base fluid. The thermophysical properties of these materials are presented in Table 2. In this study, the effects of two solid volume fractions of SWCNT and MWCNT in water and Kerosene on the flow and heat transfer characteristics have been investigated. Thermal characteristics of the studied nanofluids are presented in Tables 2 and 3^59^. In the current investigation, 3D flow is assumed to be incompressible, Newtonian, and two-phases. The fluid has a uniform velocity at the entrance of the microchannel.

**Table 2:** Thermophysical properties of different base fluids and CNTs.

Properties	Base Fluid		Nanoparticles	
	Water	Kerosene	SWCNT	MWCNT
ρ (kg/m^3^)	997.0	783.0	2,600	1,600
c_p_ (J/kg. K)	4.179	2,090	425.0	796.0
k (W/m. K)	0.613	0.145	6,600	3,000

**Table 3:** Thermophysical properties of nanofluids with the solid volume fraction of SWCNT and MWCNT.

Properties	SWCNT (0.04)		SWCNT (0.08)		MWCNT (0.04)		MWCNT (0.08)	
	Water	Kerosene	Water	Kerosene	Water	Kerosene	Water	Kerosene
ρ (kg/m^3^)	1,061	855.0	1,125	928.0	1,021	815.0	1,045	848.0
c_p_ (J/kg. K)	3811	1889	3485	1717	3968	1989	3766	1895
k (W/m. K)	1.051	0.2740	1.528	0.4100	1.011	0.2650	1.444	0.3900

### Governing equations and numerical solution method

In the numerical simulation, the flow regime is considered turbulent. Nanofluid properties are assumed to be constant and temperature independent. A solid-liquid suspension is modeled using the two-phase mixture model. Furthermore, the nanofluid is assumed as a Newtonian fluid. Diffusion effects are also ignored, and the effects of gravity acceleration are negligible. Based on these assumptions, the continuity, momentum, and energy equations can be written as^60^:

Continuity equation:

1 $$\nabla .({\rho }_{m}{\overrightarrow{v}}_{m})=0$$

where ${\overrightarrow{v}}_{m}$ is defined as follows:

2 $${\overrightarrow{v}}_{m}=\mathop{\sum }\limits_{k=1}^{n}\frac{{\alpha }_{k}{\rho }_{m}{\overrightarrow{v}}_{k}}{{\rho }_{m}}$$

where *ρ*_*m*_ is the density of the suspension:

3 $${\rho }_{m}=\mathop{\sum }\limits_{k=1}^{n}{\alpha }_{k}{\rho }_{k}$$

Momentum equation:

4 $$\nabla .({\rho }_{m}{\overrightarrow{v}}_{m}{\overrightarrow{v}}_{m})=-\,\nabla P+\nabla .[{\mu }_{m}(\nabla {\overrightarrow{v}}_{m}+\nabla {\overrightarrow{v}}_{m}^{T})]+{\rho }_{m}\overrightarrow{g}+\nabla .(\mathop{\sum }\limits_{k=1}^{n}{\alpha }_{k}{\rho }_{k}{\overrightarrow{v}}_{dr}{\overrightarrow{v}}_{dr,k}$$

where n and *μ*_*m*_ represent the number of phases and viscosity of the mixture, respectively.

Energy equation:

5 $$\frac{\partial }{\partial {\rm{x}}}\mathop{\sum }\limits_{k=1}^{n}({\alpha }_{k}{\rho }_{k}{E}_{k})+\nabla .\sum ({\alpha }_{k}{\overrightarrow{v}}_{k}({\rho }_{k}{E}_{K}+p))=\nabla .({k}_{eff}\nabla T)+{S}_{E}$$

where ***k***_***eff***_ is the effective thermal conductivity.

The turbulence equations of standard Κ–ε are applied for the simulations. The standard model of κ -ε is as follows^61^:

6 $$\frac{\partial }{\partial {X}_{i}}(\rho \,k\,{u}_{i})=\frac{\partial }{\partial {X}_{j}}\left((\mu +\frac{{\mu }_{t}}{{\sigma }_{k}})\frac{\partial k}{\partial {X}_{j}}\right)+{G}_{k}-\rho \,\varepsilon $$

7 $$\begin{array}{c}\frac{\partial }{\partial {X}_{i}}(\rho \,\varepsilon \,{u}_{i})=\frac{\partial }{\partial {X}_{j}}\left((\mu +\frac{{\mu }_{t}}{{\sigma }_{\varepsilon }})\frac{\partial \varepsilon }{\partial {X}_{j}}\right)+{C}_{1\varepsilon }\,\frac{\varepsilon }{k}\,{G}_{k}-{C}_{2\varepsilon }\frac{{\varepsilon }^{2}}{k}\,\rho \\ {G}_{k}=(-\rho \,\overline{{u}_{i}^{/}{u}_{j}^{/}})\frac{\partial {u}_{j}}{\partial {X}_{i}}\end{array}$$

The ***G***_***k***_ is the perturbation energy production, ***σ***_***k***_ is effective Prantdl for turbulence energy, and ***σ***_***k***_ is turbulence energy loss. ***C***_***1ε***_ and ***C***_***2ε***_ are constants and ***μ***_***t***_ is perturbation viscosity which is defined as:

8 $${\mu }_{t}={C}_{\mu }\,\frac{{k}^{2}}{\varepsilon }\,\rho \,$$

where ***C***_***μ***_ is a constant value which is equal to 0.09 for C_1ε_ = 1.44, C_2ε_ = 1.92, σ_k_ =1 and σ_ε_ = 1.3^62^.

In the current numerical simulation, SIMLPLE method is used for the velocity and pressure coupling, the second-order upwind schemes are imposed on all the transport equations, and the convergence criteria are considered 10^−6^.

The following equations have been used to calculate the local Nu number along the microchannel walls:

9 $$N{u}_{ave}=\frac{q{\prime\prime} {D}_{h}}{{k}_{f}({T}_{w}-{T}_{m})}$$

where the values of ***T***_***w***_ and ***T***_***m***_ refer to the average temperature of the wall and bulk temperature of the fluid^63^, respectively, and *q*″ is the heat flux applied to the surfaces of microchannels.

The following equation is used for calculating the fanning friction factor^64^:

10 $$f=2\Delta P\frac{{D}_{h}}{L}\frac{1}{\rho {{u}_{in}}^{2}}$$

where Δ*P*,*L* and *u*_*in*_ refer to pressure drop, the channel length, and inlet velocity of the fluid in the microchannels, respectively. The pumping power is calculated from the following equation^64^:

11 $$Pp=A\,{u}_{in}\,\Delta P$$

where ***A*** is the inlet cross-sectional area of the microchannel. To evaluate the heat transfer performance of the microchannel, the performance evaluation criterion (PEC) is defined as follows^65^:

12 $$PEC=\frac{(N{u}_{ave}/N{u}_{ave,\varphi =0})}{{(f/{f}_{\varphi =0})}^{(1/3)}}$$

where *Nu*_*ave*_, *Nu*_*ave,φ*=0_ represent the Nu number of the nanofluid and base fluid (water) at the same Re number, respectively. Moreover, *f*, *f*_*φ*=0_ represent the nanofluid and base fluid average friction coefficient number at the same Re number, respectively.

### Mesh independence

**Figure 3:**
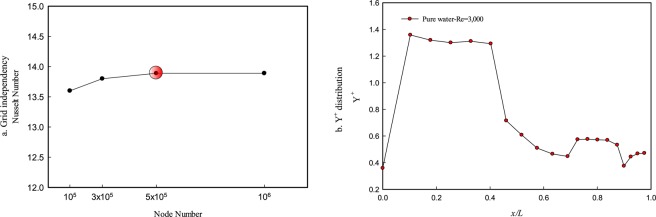
Grid independence and Y+ distribution.

In order to guarantee the minimum effect of grid number on the numerical results, different grid sizes have been applied in the computational domain. Fluid flow and heat transfer have been simulated at the Re = 200, and the result is presented in Fig. 3a. As can be seen, when the total number of grid increase from 500,125 to 1,053,563, the change of Nu number is less than 2%. Thus the rest of the simulations have been done with a 500,125 grid number. In the conducted simulation, standard wall function is used, and Y+ distribution is presented in Fig. 3b. As can be seen, the maximum value of Y^+^ is 1.4, that is consistent with the used turbulence model.

### Validation of numerical results

**Figure 4:**
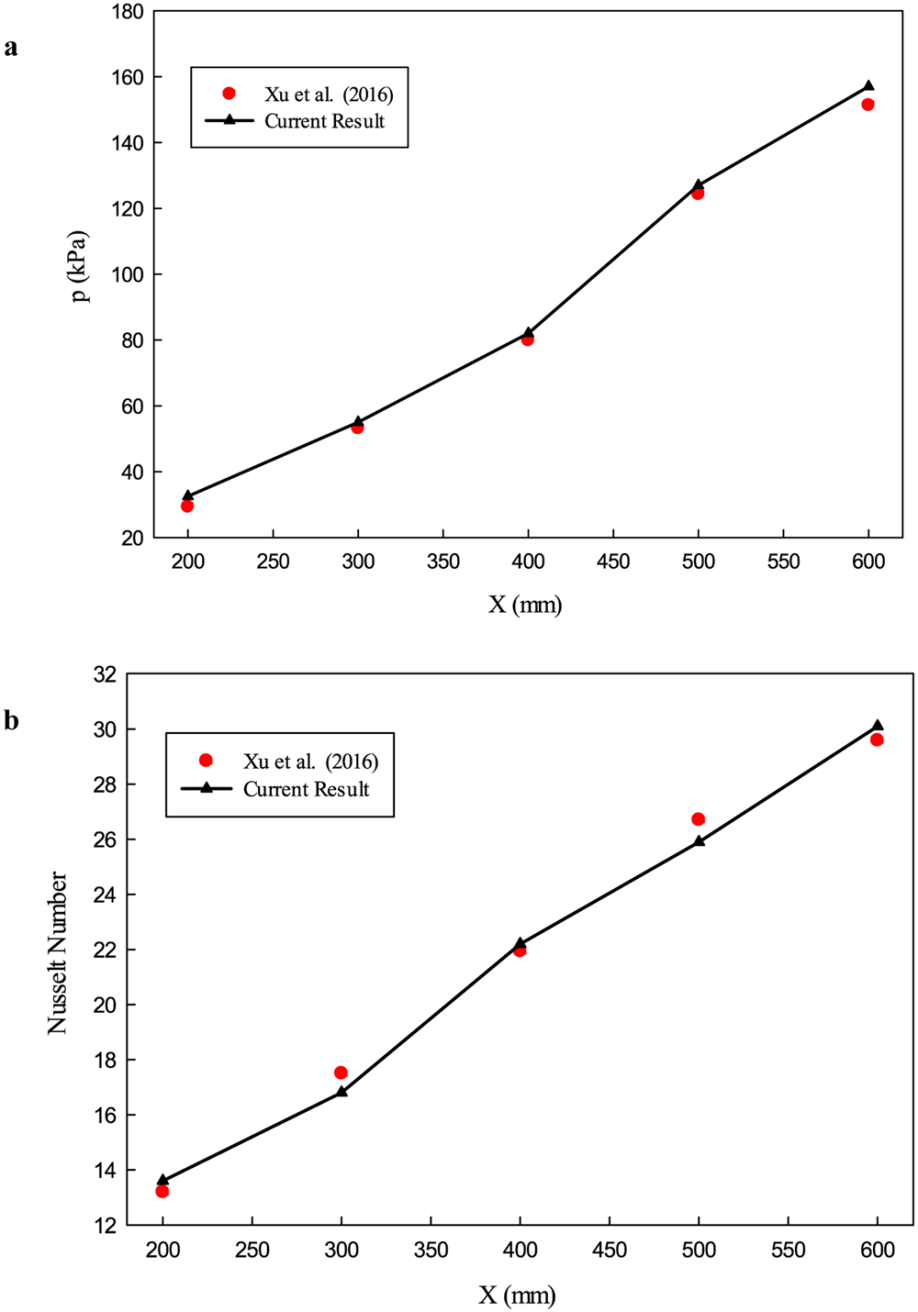
The results of the validation study; comparison between the current study and Xu *et al*.^54^ for (**a**) pressure drop and (**b**) average Nu number.

In order to ensure the accuracy of the numerical results, the numerical results have been compared with the experimental results presented by Xu *et al*.^54^. They investigated the fluid flow and heat transfer in the fractal silicon microchannel. In Fig. 4, the comparison of the Nu number and pressure loss has been presented. As can be seen, the current result has excellent compatibility with experimental data that show the accuracy of the numerical method employed in the presented study.

## Results and discussion

**Figure 5:**
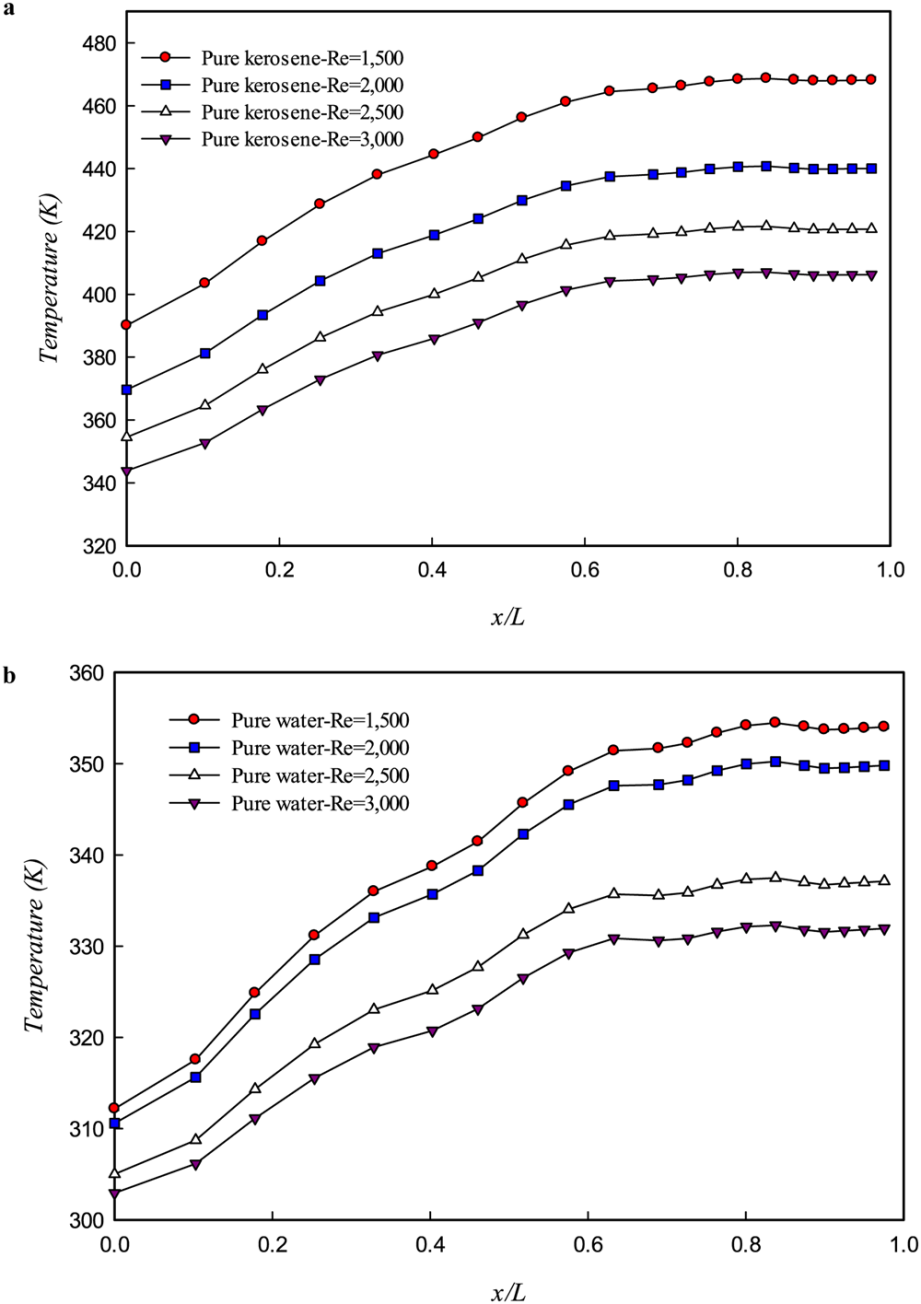
Wall temperature distribution at different Re numbers for (**a**) kerosene (**b**) water.

Figure 5 shows the effects of Re number on the wall temperature distribution along path L_1_, L_2_, L_3_, and L_4_ for pure water (a) and pure kerosene (b). As can be seen, the wall temperature increased along the path. In other words, the temperature difference between the wall and fluid decreased along the path, which leads to reducing the heat transfer rate. The comparison of the wall temperature distribution for pure water and kerosene shows that the maximum wall temperature when working fluid is water is less than that of the kerosene. Therefore, the thermophysical properties of pure water are more suitable for use in a fractal microchannel. Kerosene in the same geometry and Re numbers has less heat transfer capability compared to pure water. As can be seen in Fig. 5, the wall temperature of the inlet is lower than the temperature of the outlet; this means that maximum temperature accrues at the last branch (L4: x/L > 0.8). The reason for this phenomenon is the high flow rate and high local Re number in the main branch and the decrease of mass flow rate along the path by dividing the flow between the branches that reduce the heat transfer and increasing the wall temperature.

Moreover, the effects of the Re number on the temperature distribution can also be analyzed based on Fig. 5. Increasing fluid velocity leads to increasing the heat transfer coefficient. Moreover, increasing the cooling fluid’s development length leads to postponing the thermal development. Due to this behavior, the temperature of the walls has been significantly reduced by increasing the velocity of the fluid; for water at Re = 1,500, the maximum temperature is 354 K, whereas at Re = 3,000 maximum temperature is 332 K.

**Figure 6:**
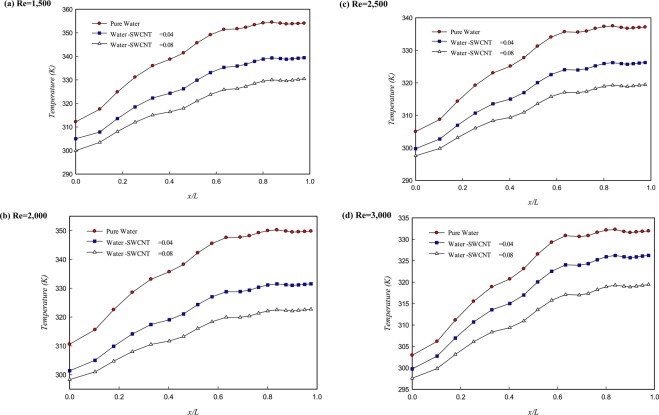
Wall temperature distribution at different volume fractions of water/SWCNT nanoparticles at different Re numbers.

The effects of adding the SWCNT nanoparticles to water in solid volume fractions 0, 0.04, and 0.08 and at the Re numbers ranging from 1,500–3,000 on the wall temperature distribution have been presented in Fig. 6. Adding the solid nanoparticles enhances the thermophysical properties such as thermal conductivity of the cooling fluid that leads to increase the heat transfer, as can be seen in Fig. 6. It is noteworthy to note that increasing fluid velocity and volume fraction of solid nanoparticles leads to decreasing the temperature gradient along the path. As a result, the wall temperature distribution will be uniform, and its maximum temperature reduced.

**Figure 7:**
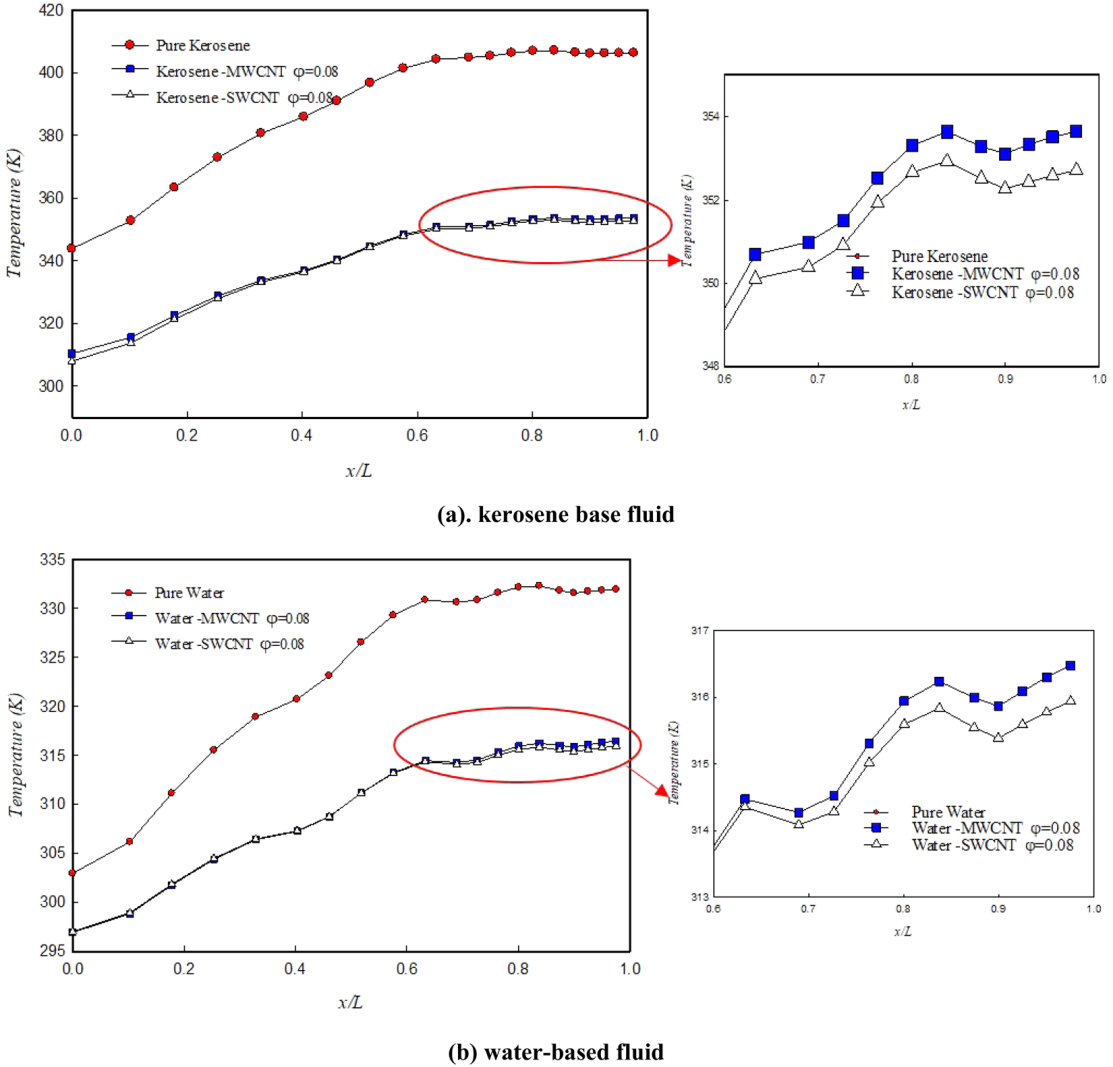
Variations of the wall temperature distribution for SWCNT and MWCNT nanoparticles in water and kerosene at Re number of 3,000.

The effect of solid volume fraction of SWCNT and MWCNT nanoparticles on the wall temperature distribution on the kerosene (a) and water (b) as working fluid at the Re number of 3,000 have been presented in Fig. 7. The qualitative process of temperature changes along the fractal microchannel is similar for water and kerosene. But what makes the quantitative changes in reducing the temperature of the walls of channels is the type of fluid and nanoparticles’ thermophysical properties. As can be seen in Fig. 7, in the same conditions, such as the same solid volume fraction and Re number, the wall temperature reduction by using the water-based nanofluid is more significant than that of the kerosene-based nanofluid. Furthermore, the SWCNT nanoparticle has a better performance than the MWCNT nanoparticle.

**Figure 8:**
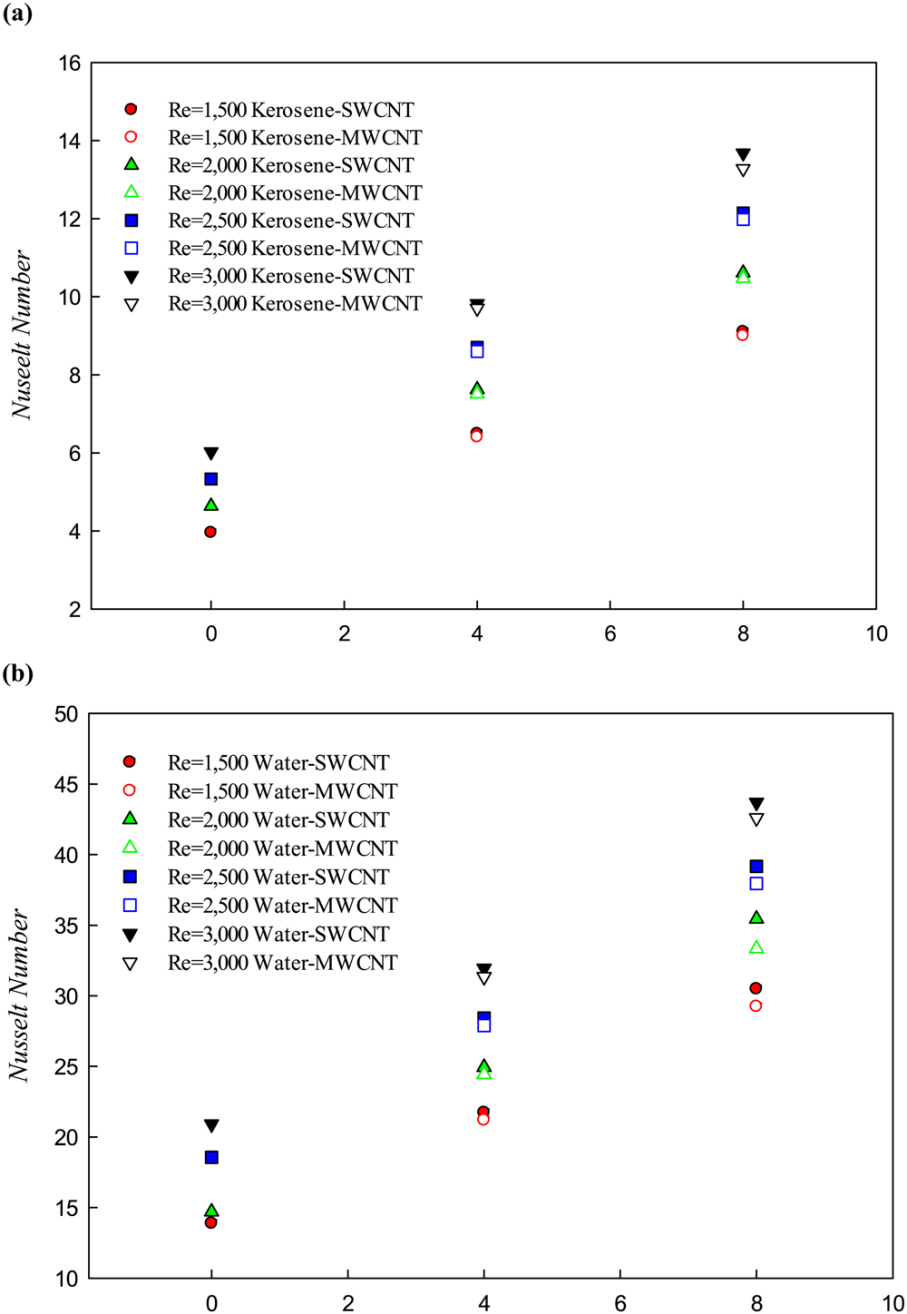
The variations of Nu number with respect to the solid volume fraction of SWCNT and MWCNT nanoparticles in water and kerosene at different Re numbers.

The average Nu number with respect to solid volume fraction at different Re numbers for kerosene and water-based nanofluid have been presented in Fig. 8. It should be noted that the geometrical structure of the microchannel and the path for all the cases presented in Fig. 8 are identical. The difference between the behaviors (Nu number) corresponds to the base fluid and the solid volume fractions. Comparing between the Nu number of the two base fluid (water and kerosene) shows that using water as a base fluid has greatly affected the rate of heat transfer so that in the same nanoparticle volume fraction and Re numbers, the average Nu number of water-based nanofluid (Fig. 8b) has increased approximately 4 times in comparison with the kerosene-based nanofluid (Fig. 8a). The use of the SWCNT nanoparticle in comparison to the MWCNT nanoparticle for both the base fluids has greatly differentiated the Nu number increase, which is more noticeable by growing the Re number and the solid volume fraction. On the other hand, the use of nanoparticles in both the base fluids at the higher Re numbers resulted in more enhancement in the Nu number. It appears that the use of nanoparticles in high Re numbers can considerably improve the heat transfer performance compared to lower Re numbers.

**Figure 9:**
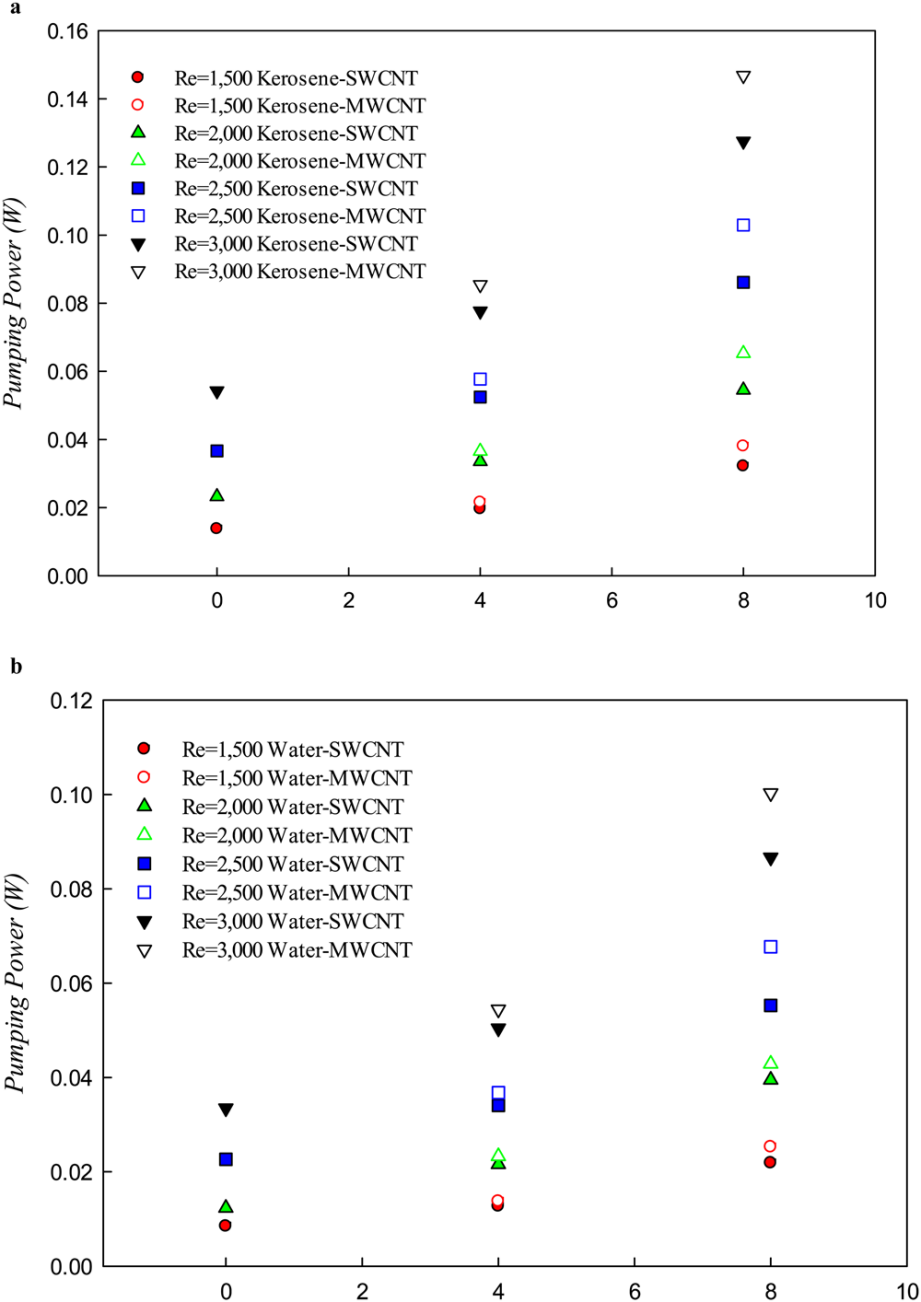
The variations of pumping power with respect to the solid volume fraction of SWCNT and MWCNT nanoparticles in water and kerosene at different Re numbers.

Figure 9 presents the variations of the pumping power with respect to the solid volume fraction of the SWCNT and MWCNT nanoparticles in the base fluids (water and kerosene) at different Re numbers. In addition to geometrical parameters, the pumping power also depends on thermophysical properties such as density, dynamic viscosity, and the inlet velocity. As can be seen, when the working fluid is kerosene, the pumping power is more than when the working fluid is water. By adding the nanoparticles to the base fluid, the density and the viscosity of the cooling fluid increased, which significantly increased the pumping power for both nanofluids with increasing volume fraction. In fact, by adding the nanoparticles to the base fluid, which leads to increasing the density and viscosity of the cooling fluid, the movement of the nanofluid in the fractal microchannel needs more pumping power. Increasing the pumping power will be more significant by increasing the nanoparticle volume fraction at higher Re numbers. Although the density and viscosity of the kerosene-based nanofluid are lower than water-based nanofluid at the same nanoparticle volume fraction, at the same Re numbers, the pumping power of kerosene-based nanofluid compared with the water-based nanofluid was significantly increased. The reason for this behavior is due to an increase in the ratio of viscosity to the density of the base fluid, which in the calculation of the Re number at the same hydraulic diameter value leads to an increase in the inlet velocity of the nanofluid. As shown in Fig. 9, the MWCNT nanofluid leads to having higher pumping power than the SWCNT nanoparticle in the two studied base fluids.

**Figure 10:**
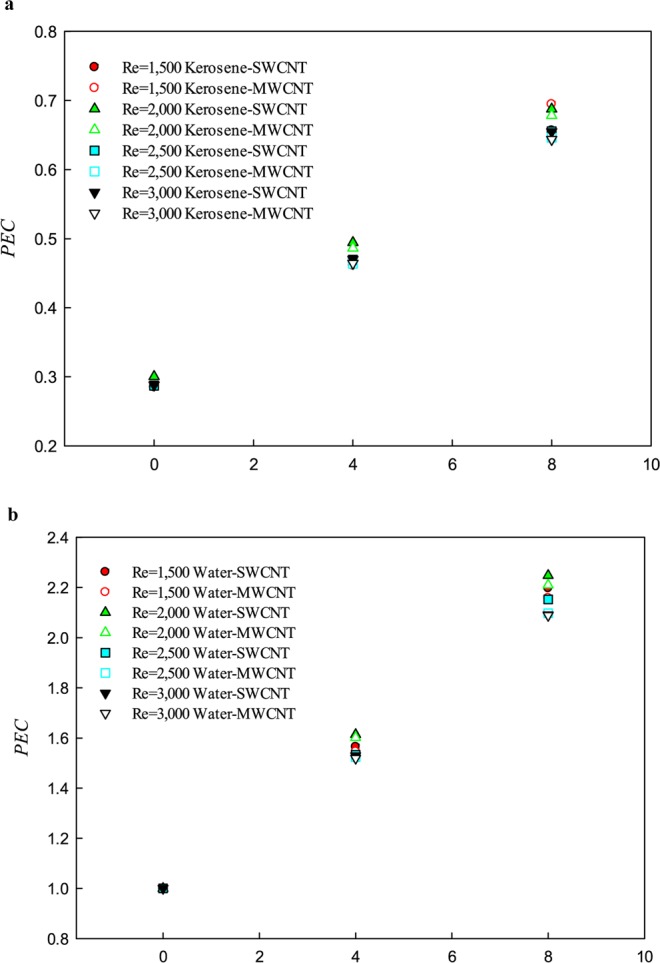
PEC in terms of volume fraction of SWCNT and MWCNT nanoparticles in the water and kerosene at different Re numbers.

Figure 10 shows the PEC with respect to the solid volume fraction of SWCNT and MWCNT nanoparticles in the two studied base fluids (water and kerosene) at the Re numbers of 1,500, 2,000, 2,500, and 3,000. The PEC is the ratio of increasing the Nu number to the increase in friction coefficient for each solid volume fraction compared to the base fluid. The variations in the PEC are affected by the heat transfer process, and the friction coefficient that can lead to different behaviors in the studied Re numbers. Factors like the addition of nanoparticle volume fractions, fluid velocity, and fluid type affect the heat transfer and flow field. Depending on the method used to increase the heat transfer, and based on the increment of friction coefficient, the behavior of the PEC can be different. As shown in Fig. 10, the addition of nanoparticle increases the PEC, which is due to an increase in the Nu number. As can be seen in Fig. 10, at the same Re number and the same solid volume fraction, the PEC for the water-based nanofluid is approximately 3 times greater than that of the kerosene-based nanofluid.

Moreover, the highest value of the PEC is found in the solid volume fractions of 4% and 8% at the Re number 2,000. Figure 10 shows that with increasing the Re number, the effects of increasing the friction coefficient is more than that of the Nu number, so that in the Re number of 3,000, at both the solid volume fractions (4 and 8%), the lowest value of the PEC is obtained. The analysis of the PEC for the kerosene-based nanofluid shows that the use of this nanofluid for cooling applications in the fractal silicon microchannel is not appropriate under the studied conditions from the engineering and economics viewpoint, but the use of the water-based nanofluid is recommended in all the studied conditions.

**Figure 11:**
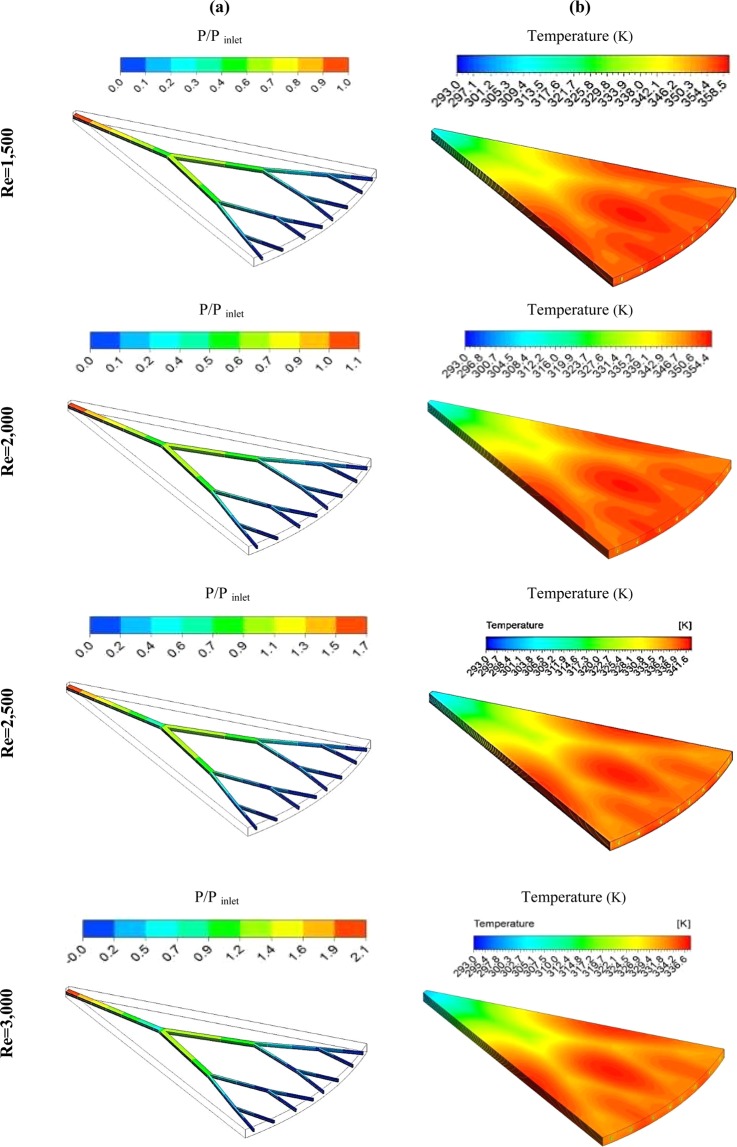
The variations of (**a**) pressure ratio and (**b**) static temperature distribution at different Re numbers for pure water.

Figure 11 presents the pressure distribution (11a) and static temperature distribution (11b) at different Re numbers for pure water. As it was observed, increasing the fluid velocity (Re number), the heat transferred from the wall to the fluid inside the fractal microchannel has improved, and the wall temperature has decreased. The effect of decreasing temperature with increasing fluid velocity in the outlet sections of each branch has created a distinct difference in temperature distribution. According to the temperature distribution, at the beginning regions of the fractal microchannel, the heat transfer is always maximized due to the high-temperature difference between the fluid and the silicon microchannel, and the temperature in these regions is close to the fluid inlet temperature. The use of the fractal structure is compensated for the heat transfer loss and the creation of heat transfer areas. By increasing the number of fractal tree branches, the temperature distribution in these areas becomes more uniform, and it prevents the formation of extreme temperature gradients. Moreover, as the Re number increases, the thermal gradients are eliminated, especially at the outlet section. However, the pressure drop enhances with increasing fluid velocity. The pressure drop is also increased by decreasing the diameter of the microchannel, and this behavior is more noticeable at the branches near the outlet section. Regarding the behavior of the pressure contours in Fig. 11, the increase in the Re number leads to maximizing the pressure loss, and in the channels with a smaller diameter, the pressure loss is more than the other channels.

**Figure 12:**
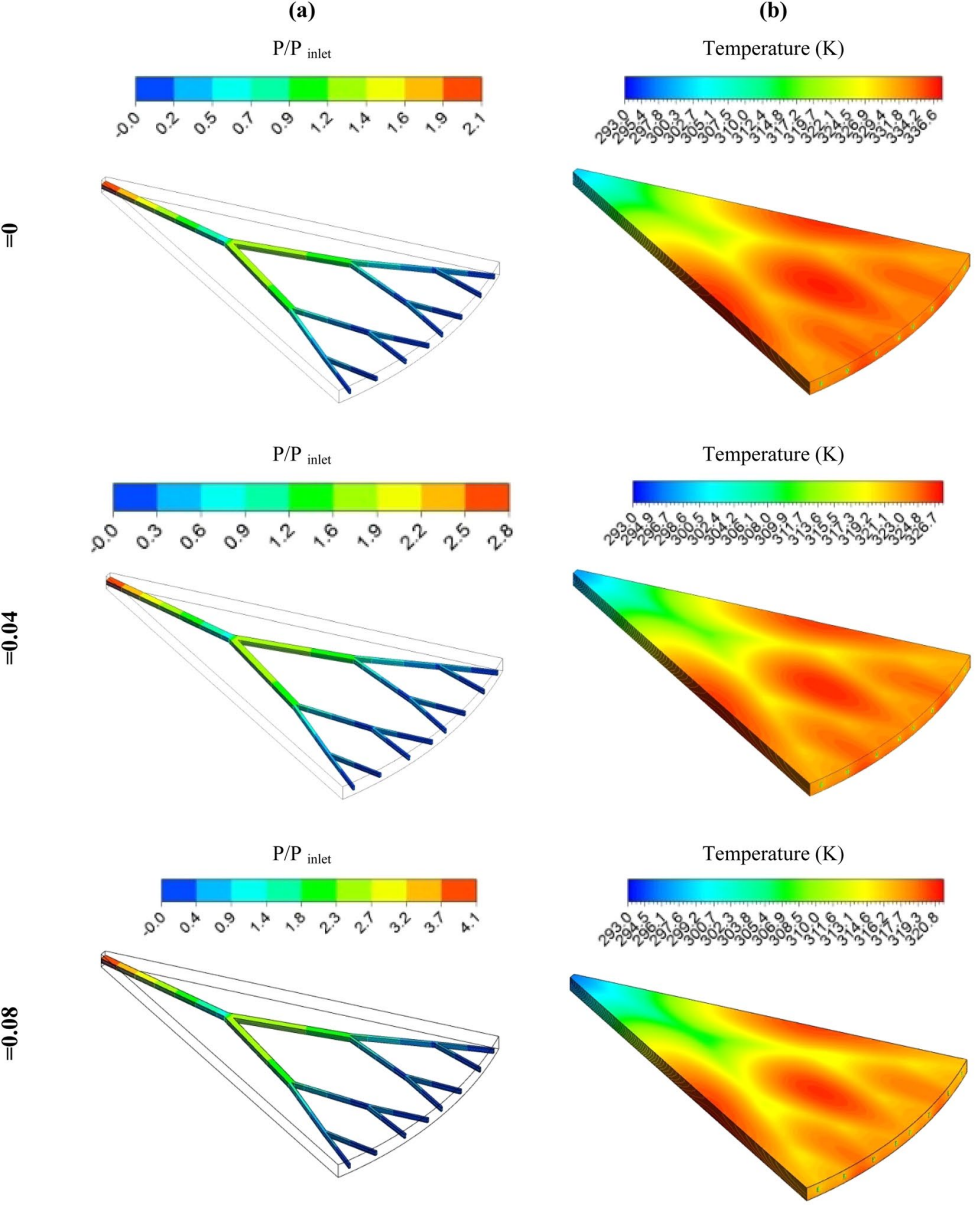
Pressure ratio (P/Pin) (**a**) and static temperature distribution (**b**) at Re number of 3,000 and SWCNT nanofluid in different volume of fraction.

In order to investigate the effects of adding the nanoparticles in different solid volume fractions in the water on the pressure distribution ratio (P/P_in_) (a) and the static temperature distribution (b) at Re number of 3,000 for three solid volume fraction of the SWCNT have been presented in Fig. 12. As can be seen, the temperature gradients have been eliminated by increasing the volume fraction of nanoparticles, and the uniform temperature distribution has been achieved in the fractal silicon microchannel. In some areas where no fluid flow paths exist, hot areas have been witnessed that can be improved by using more tree-like structures. The presence of the nanoparticle with the higher solid volume fractions will significantly improve the thermophysical properties, especially the thermal conductivity of the cooling fluid that accelerates the heat transfer and causes uniformity of the temperature distribution of the walls. On the other hand, the addition of the nanoparticles in the base fluid increases fluid properties such as density and viscosity, which leads to having a higher pressure drop. Increasing the heat transfer is the advantage of using nanofluid, and increasing pressure drop is the main disadvantage, which the PEC is the criterion for its performance evaluation. According to the results, the PEC of the water-based SWCNT nanofluid is more than the other investigated cases.

**Figure 13:**
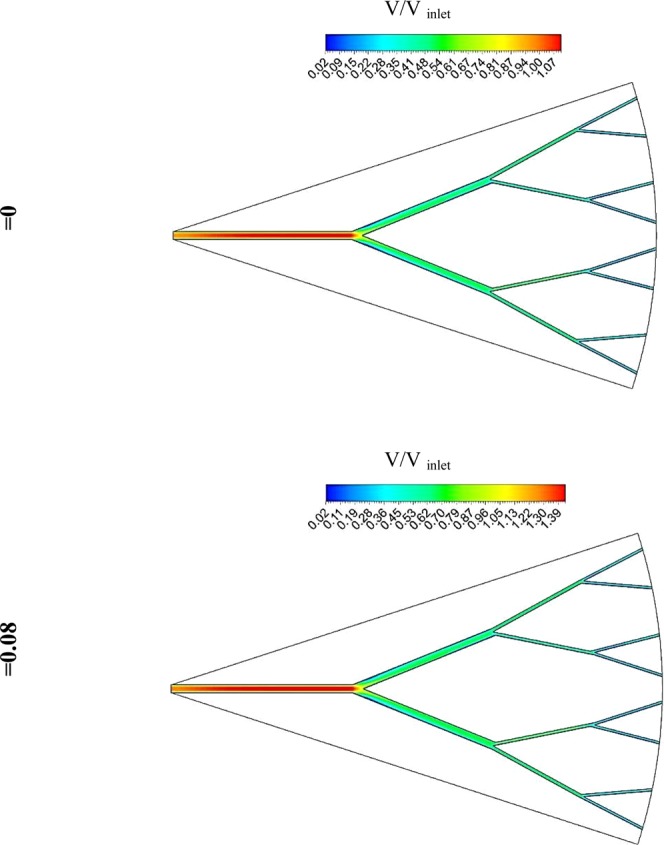
Velocity distribution at Re number of 3,000 pure water and water- SWCNT (φ = 0.08).

In Fig. 13 contour of velocity distribution when the working flow is pure water and water-SWCNT (φ = 0.08) at Re = 3,000 are shown. As seen in the main branch, velocity is maximum, and sub-branch the flow is divided, and velocity decreases. By reduction of velocity heat transfer rate decrease and wall temperature increase on a branch near the outlet.

## Conclusion

In the present numerical study, the heat transfer and fluid flow were simulated in a fractal microchannel with two working fluids of water and kerosene suing SWCNT and MWCNT nanoparticles in different solid volume fractions ranging from 0 to 8 vol. % employing finite volume method. Investigating the flow and temperature fields of the nanofluids in fractal silicon microchannel and analysis of the temperature distribution, pressure drop, Nu number, PEC, temperature, and pressure distribution at each investigated Re numbers, distinguish the current study from other similar studies. The results showed that due to the contact of cooling fluid with the hot walls of the microchannel, fluid temperature increases that cause reducing the heat transfer between hot walls and working fluid. This behavior has created a significant static wall temperature gradient. As the Re number increases, the heat transfer coefficient increases, which leads to decreasing the wall temperature. The results showed that in both the studied nanofluids, the temperature drop across the fractal microchannel wall is significant, and the MWCNT nanoparticle is more suitable than the SWCNT nanoparticle. Although adding the nanoparticles lead to improving the heat transfer, they significantly increase the pumping power by increasing the density and viscosity of the cooling fluid. According to the obtained results, the addition of the studied nanoparticles increases the PEC, which is caused by the increase of the Nu number. The results of the current study showed that the SWCNT-water nanofluid has the maximum PEC, and using this nanofluid in a fractal microchannel is highly recommended.
